# Causal Mechanistic Reasoning as a Tool to Explore Medical Students’ Predictions of Pharmacology Phenomenon: Connecting Core Concepts with Clinical Applications

**DOI:** 10.1007/s40670-025-02432-6

**Published:** 2025-06-04

**Authors:** Rosalyn R. Bloch, Keenan Noyes, Nathan Bautista, Carolina B. Restini

**Affiliations:** 1 College of Osteopathic Medicine, Michigan State University, East Lansing, MI USA; 2 Corewell Health Farmington Hills, Farmington Hills, MI USA; 3 Department of Physiology, Michigan State University, East Lansing, MI USA; 4 Department of Pharmacology and Toxicology, College of Osteopathic Medicine (Macomb University College – MUC; and Detroit Medical Center – DMC; and East Lansing), Michigan State University, 44575 Garfield Road, Building UC4, Clinton Township, MI 48038 USA

**Keywords:** Pharmacology, Medical education, Core concepts, Causal mechanistic reasoning, Adverse effect

## Abstract

**Introduction:**

Prior research in education has identified that causal mechanistic reasoning (CMR) can enhance understanding of causal relationships and support the construction of explanations and predictions. However, the literature lacks information about how CMR is used among medical students or in pharmacology education. This study investigated how medical students utilize CMR to predict and explain adverse drug effects (ADE) as a pharmacological phenomenon.

**Methods:**

Pre-clerkship osteopathic medical students enrolled at a large American university were asked to predict and explain their reasoning related to adverse effects caused by SGLT2 inhibitors. Their responses guided the development of a coding scheme to characterize the degree to which students used CMR. Pearson’s chi-squared tests were applied to analyze the presence and strength of the relationships between overall ADE predictions and the type of CMR used.

**Results:**

Sixty-seven percent of the students (*N* = 88) correctly identified urogenital infections as a possible ADE caused by SGLT2 inhibitors; however, only 25% provided a fully causal mechanistic account. However, we identified a significant association of large effect size between using CMR and correctly predicting the ADE (*χ*^2^ = 56.129, *p*-value < 0.001, Cramer’s *V* = 0.799).

**Conclusion:**

CMR can be a useful tool for supporting medical students’ understanding of pharmacological phenomena and solidifying students’ learning toward an effective application in future clinical practice. This research highlights how more integrative, mechanism-focused curricula may be a promising area of future research in pharmacology education research.

**Graphical Abstract:**

How students employ mechanistic reasoning to connect foundational biomedical sciences (e.g., physiology, microbiology, biochemistry) with core pharmacological concepts, such as pharmacodynamics, to think through the potential adverse effects of a drug class (SGLT2 inhibitor). Causal mechanistic reasoning (CMR) can be used to understand how students use their knowledge of the underlying entities to explain a phenomenon and address pharmacology-specific questions.

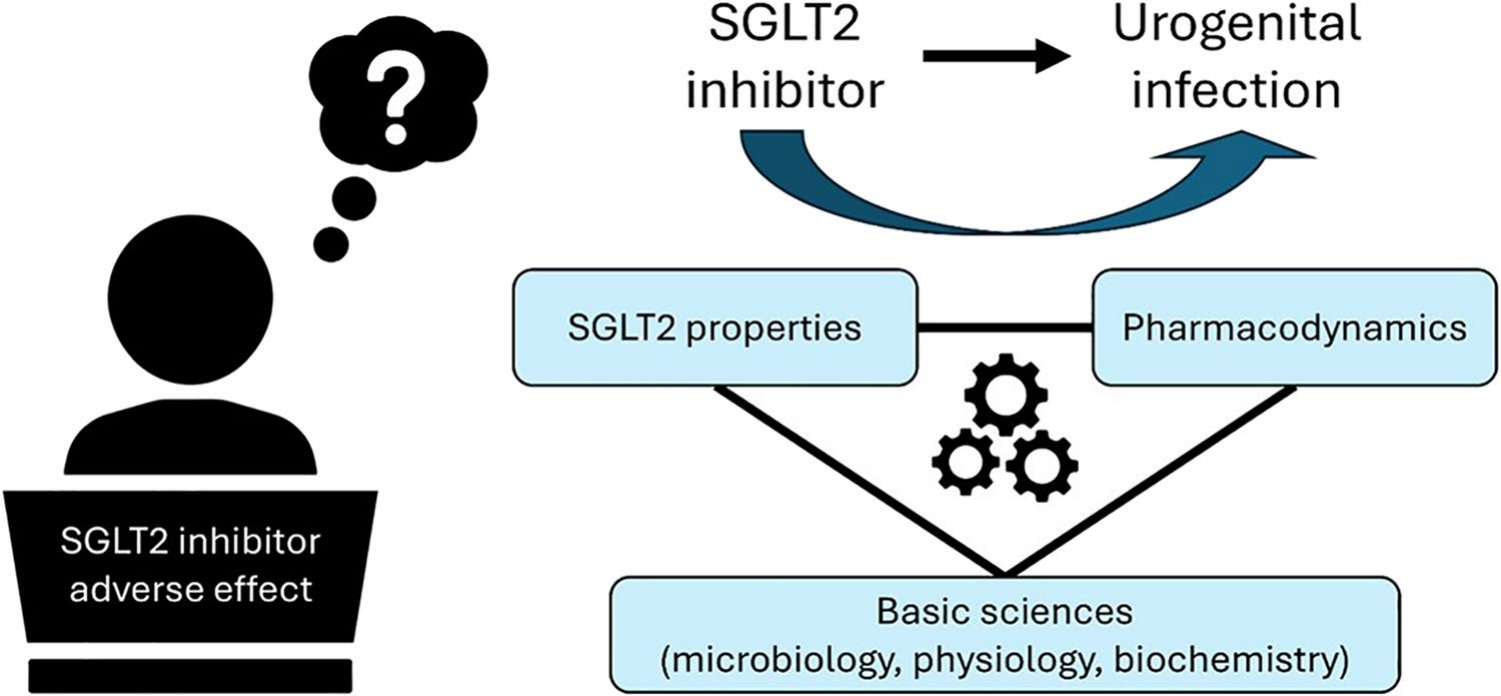

**Supplementary Information:**

The online version contains supplementary material available at 10.1007/s40670-025-02432-6.

## Introduction

### The Importance of Pharmacology in Medicine and Medical Education

Pharmacology, the science of the dynamic interactions between drugs and the body, is a fundamental pillar of medical practice [1]. Mastering pharmacological principles is essential for effective integrated healthcare as it allows one to safely navigate the clinical complexities of diagnosing and treating patients. Therefore, comprehension, analysis, and application of basic concepts are critical for the training of not just clinical pharmacologists but *all* physicians [1–3]. However, current research indicates that junior physicians and medical students have a poor understanding of pharmacology [4–12]. This is concerning as a lack of foundational pharmacological knowledge is a key factor contributing to medication errors [6, 9, 13, 14]. Even though medication errors are among the most common medical errors, they are mostly preventable through appropriate medication reconciliation, which is founded on pharmacology foundational knowledge to integrate clinical aspects of therapeutics [15–19]. However, the lack of knowledge on fundamental concepts in pharmacology has a significant negative impact on patients’ therapeutics, such as the length of stay and hospital strain [20–23]. This underscores the growing calls to improve pharmacology education [2, 4, 24].

Historically, pharmacology education in medical schools has been conducted through a discipline-focused and lecture-centric method [2, 9]. In recent years, advancements in medical education have focused on (1) improving the integration of the basic and clinical sciences and (2) incorporating more student-centered instructional techniques. Such changes arose in response to prior research uncovering that existing approaches failed to adequately prepare medical students for therapeutic applications in clinically relevant scenarios [25]. Integrating the medical education pre-clerkship curricula aims to more explicitly connect the basic (foundational) biomedical sciences to clinical applications of medical content [26, 27]. As a result, pharmacology is no longer covered as a standalone unit in such systems; instead, it must be covered throughout each organ system. This design has unintentionally created greater challenges for pharmacology education as instruction centered around pharmacology core concepts may be reduced unless clearly defined as a key discipline that bridges basic sciences to clinical application in medical education broadly [3, 9].

Implementing student-centered learning methods in medical education [28] has prompted a more critical examination of curriculum content to enhance clinical relevance in pre-clerkship courses. These teaching methods have been positively received by students learning pharmacology [29–31]. Crowley et al. comprehensively reviewed pedagogical strategies in pharmacology across global health professions programs, identifying didactic lectures, team-based learning (TBL), problem-based learning (PBL), case-based learning (CBL), simulation-based learning, flipped classroom, and gamification as the commonly used pedagogical approaches. These strategies integrate pharmacological principles with basic and clinical sciences, fostering knowledge acquisition and medication management skills through clinical case discussions and active learning [32].In addition to active learning methods, social pedagogy is another teaching model that has been used in medical education which emphasizes the social nature of learning to provide structured support and scaffolding to enhance learner-led exploration and collaboration [33].

As demonstrated through educational investigations, data on pharmacology education research aligns with positive results from other basic science disciplines regarding the impact of active learning on students, including engagement and rates of passing [34, 35]. However, while students experienced positive outcomes through active learning (e.g., higher test scores and increased satisfaction), reports also indicated a lack of training in therapeutics and inadequate foundational drug knowledge, underscoring the need for enhanced educational strategies to improve medical students’ comprehension of basic pharmacological principles [36, 37]. Additional work in pedagogical techniques must be done to reflect on the content taught using active learning techniques and the resulting impacts on student knowledge development [38]. That is, we must go beyond just thinking about *how* students are taught to consider *what* they are taught.

One example of this is the recent effort to identify the core concepts of pharmacology [39–42]. Core concepts provide a way to reflect on the critical ideas within a discipline. The core concepts can also interlink to explain a biological phenomenon [42]. Foundational concepts within pharmacology include the mechanism of action (pharmacodynamics), pharmacokinetics, concepts of drug interactions, and adverse drug events and reactions. This is closely related to a broader form of reasoning useful to advancing pharmacology education: mechanistic reasoning.

### Mechanistic Reasoning in Science Education

Mechanisms are fundamental to science [43–45]. The presence of a mechanism indicates that a relationship is causal and not just an unrelated correlation [46]. That is, a mechanism provides the link connecting two otherwise disparate events. This is why mechanisms are sought after in science—they provide the necessary information that both proves the existence of a causal relationship and, through knowledge of the mechanism, provides a way to manipulate that relationship. For example, if one understands the mechanism, they can make robust predictions about how the mechanism can be changed to produce a certain outcome in the resulting phenomenon.

While several scholars have defined the philosophical concept of a mechanism in science, Illari and Williamson [45] propose a consensus definition based on the shared features of those descriptions: “[a] mechanism for a phenomenon consists of entities and activities organized in such a way that they are responsible for a phenomenon” [45]. In this definition, the “entities” are the relevant components of a system, along with their properties. “Activities” are the verbs—what the entities do (as a result of their properties) that gives rise to the phenomenon. Education researchers have adapted this definition to incorporate literature from cognitive science and about how people learn to create practical tools for exploring student thinking [47–50]. In this study, we primarily rely on the framework outlined by Krist et al., which identifies three epistemic heuristics (i.e., mental shortcuts) necessary for a learner to engage in mechanistic reasoning [49]. First, the learner must step down (at least) one scalar level to consider the relevant underlying entities. Next, they must unpack the properties and behaviors (i.e., activities) of those entities. Finally, the learner must link the properties and behaviors of the underlying entities back to the phenomenon.

Mechanistic reasoning is becoming an increasingly popular area of science education research [51]. This framework has been used to explore student thinking in the context of phase changes [52–54], chemical reactions [55–60], differential gene expression and mutations [61, 62], the transfer of energy [63, 64], flow of electrical currents [65], and many others. To contextualize how mechanistic reasoning has been used to explore student thinking, we explore two studies in depth.

In the first study, Franovic et al. explored how students used mechanistic reasoning to predict and explain protein–ligand binding [66]. The students were shown two versions of a protein binding site and asked which would preferentially bind a magnesium ion. The two protein versions differed in that one version had more negatively charged amino acids than the other so that it would form a stronger attractive interaction with the positively charged magnesium ion (Mg^2+^). They developed a coding scheme which captured if students stepped down a scalar level to consider the underlying entities (atoms within the amino acid side chains), unpack the properties of those entities (charge of the atoms or polarity of the bonds), and then link entities’ properties to the phenomenon (more negatively charged binding site will more strongly attract Mg^2+^). The students were then characterized by the degree to which they engaged in mechanistic reasoning, depending on which components of a mechanistic explanation they included. Franovic et al. then used this framework to examine the relationship between engaging in mechanistic reasoning and correctly predicting the protein version Mg^2+^ would bind to [66]. They found that those who used mechanistic reasoning in their explanation were significantly more likely to make a correct prediction compared to those who provided a partially mechanistic or non-mechanistic account.

The second example comes from physiology, in which Doherty et al. use the core concept of flow down gradients to explain bulk flow in multiple biological contexts [67]. The concept of flow down gradients is that the rate of fluid movement is determined by the resistance to fluid movement and the pressure gradient; the latter is the focus of their study. They developed a coding scheme to characterize how students consider the mechanism of bulk flow. Specifically, they were interested in how students considered fluid flow not just at the system level but going underneath to consider the properties of the fluid (specifically pressure) at different parts of the system. Once they considered the differential pressure of the fluid at different points of the system (start and end), they could determine the pressure gradient, then linking the pressure gradient to bulk fluid movement. Doherty et al. then used this coding scheme to assess how students were thinking across multiple different contexts: the movement of blood through blood vessels, sap through phloem, and a generic fluid through a tube. They found that students were remarkably consistent across the scenarios. That is, if they used mechanistic reasoning in one context, they were more likely to use that mechanistic reasoning in another context as well.

These studies highlight just some ways that mechanistic reasoning can guide and inform research. The lens of mechanistic reasoning is often used to characterize how a student reasons through a specific phenomenon. This can provide insight into how students use mechanistic reasoning in a given context, but also to answer broader questions about how student learning occurs. Given the utility of this form of reasoning along with the importance of the pharmacology core concept of mechanism of action, causal mechanistic reasoning (CMR) could be a valuable tool for guiding the development of pharmacology curricula. Also, there are certainly parallels with calls to integrate the medical education pre-clerkship curricula, which aim to more explicitly connect the basic (foundational) biomedical sciences to clinical applications of medical content [26, 27]. Still, those are examining this issue broadly, and the lens of mechanistic reasoning may allow us to focus in on the most critical aspects of the underlying physiology/pathophysiology (i.e., the mechanism). However, prior to our study, limited research has investigated how students employ mechanistic reasoning to connect foundational biomedical sciences (e.g., physiology, microbiology, biochemistry) with core pharmacological concepts such as pharmacodynamics and pharmacokinetics to address pharmacology-specific questions. Aronson [68] and Tonelli and Williamson [69] discuss the role of mechanisms in medicine, but they approach the subject through a lens of philosophy of science (e.g., Illari and Williamson [45]). To bridge this gap, we drew upon the broader science education literature to analyze how students use CMR to understand and articulate the potential adverse effects of an SGLT2 inhibitor [47, 49]. We hypothesize that students using CMR to understand pharmacologic pathways can develop predictive insights regarding pharmacologic agents and exhibit a more comprehensive understanding of core concepts of pharmacology by evaluating students’ application of a drug’s adverse effects concepts to solve a clinical case.

In this sense, we explored students’ reasoning about the adverse effects of an SGLT2 inhibitor and developed a methodology to characterize students’ use of CMR. Our study was founded on three main research questions:

1. What possible adverse effect do medical students predict could be caused by an SGLT2 inhibitor?
2. How do medical students use causal mechanistic reasoning to explain the possible adverse effect of an SGLT2 inhibitor?
3. How is the use of causal mechanistic reasoning associated with correctly predicting the possible adverse effect of SGLT2 inhibitors?

## Methods

### Study Design, Recruitment of Participants, and Data Collection

We developed a cross-sectional observational analytical study including pre-clerkship osteopathic medical students from a large, public midwestern medical university who recently completed the standalone Medical Pharmacology Course (IRB approval: Study ID STUDY00007350). We informed students of the project’s purpose in an email via the institutional listserv and invited them to voluntarily participate in an anonymous survey. Participants accessed the survey via QR code or the link distributed in the email and then completed the study on the online platform Qualtrics® [70]. Consenting participants (*N* = 88) who completed the survey entered a drawing to win one of six $25 Amazon gift cards. We de-identified the responses prior to analysis.

The survey consisted of sections on demographics, study resources/strategies, and pharmacology questions (as described below). As variables for demographic analysis, we collected data on gender identity, age, year in the program, academic quintile, and previous pharmacology background. Additionally, to get a more complete profile of the students, we asked participants to indicate which resources/strategies they used to study pharmacology (Supplemental S1). These included external resources (e.g., Sketchy [71], Anki [72]), internal resources (e.g., coursepack, OnTarget [73]), and general study strategies (e.g., rewriting the learning materials, rewatching lectures).

### Prompt Design for Causal Mechanistic Reasoning Questions

**Fig. 1:**
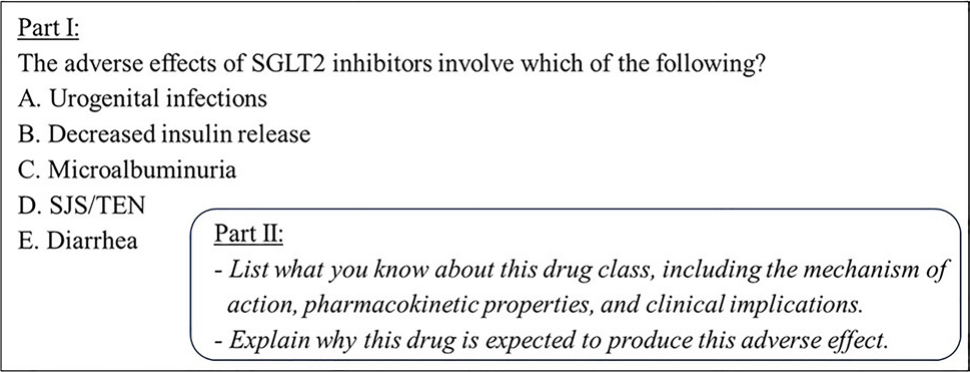
Questions designed to elicit CMR of the pharmacological phenomenon. Students answered the multiple-choice question (part I, with the correct choice being A) and then had two short-answer questions to explain their reasoning for the italicized questions (part II). SJS, Stevens-Johnson syndrome; TEN, toxic epidermal necrolysis

To elicit students’ use of CMR in a clinical context, we elaborated on a lead-in to assess potential adverse effects caused by sodium-glucose co-transporter 2 (SGLT2) inhibitors (Fig. 1). It is noteworthy that students did not receive explicit training about causal mechanistic reasoning. We designed the task to include both multiple-choice and short-answer format questions.

In part I, we asked students to select one of five possible adverse effects caused by an SGLT2 inhibitor: urogenital infections (the only correct answer), decreased insulin release, microalbuminuria, SJS/TEN, and diarrhea (Fig. 1). Subsequently, in part II, students responded to two short-answer questions. Students listed the underlying aspects of the SGLT2 inhibitors. Then, they explained why this class of drugs was expected to produce the adverse effect they chose in part I. Taken together, these questions provided evidence of students’ ability to provide a causal mechanistic account for this pharmacological phenomenon.

### Analyzing the Responses and Developing a Coding Scheme

Based on students’ responses to parts I and II (Fig. 1), we developed a coding scheme to characterize how the students engaged with CMR when explaining why an SGLT2 inhibitor would produce a chosen adverse effect. The coding scheme was founded on the definition of causal mechanistic reasoning put forth by Krist and colleagues [49], stating that a causal mechanistic response should identify and unpack the relevant properties and behaviors of the entities at a scalar level below the “observed phenomenon” and then link the underlying mechanism to the phenomenon. For our prompt, we treated the “observed phenomenon” as the adverse effect caused by the SGLT2 inhibitor (i.e., a urogenital infection) and the students’ responses to the short-answer questions (Fig. 1, part II) as a causal mechanistic explanation of this phenomenon. Therefore, we considered the evidence-based pharmacology of SGLT-2 inhibitors [74] as the “relevant properties and behaviors of the entities” CMR component, we aimed to capture in our coding scheme [49].

**Fig. 2:**
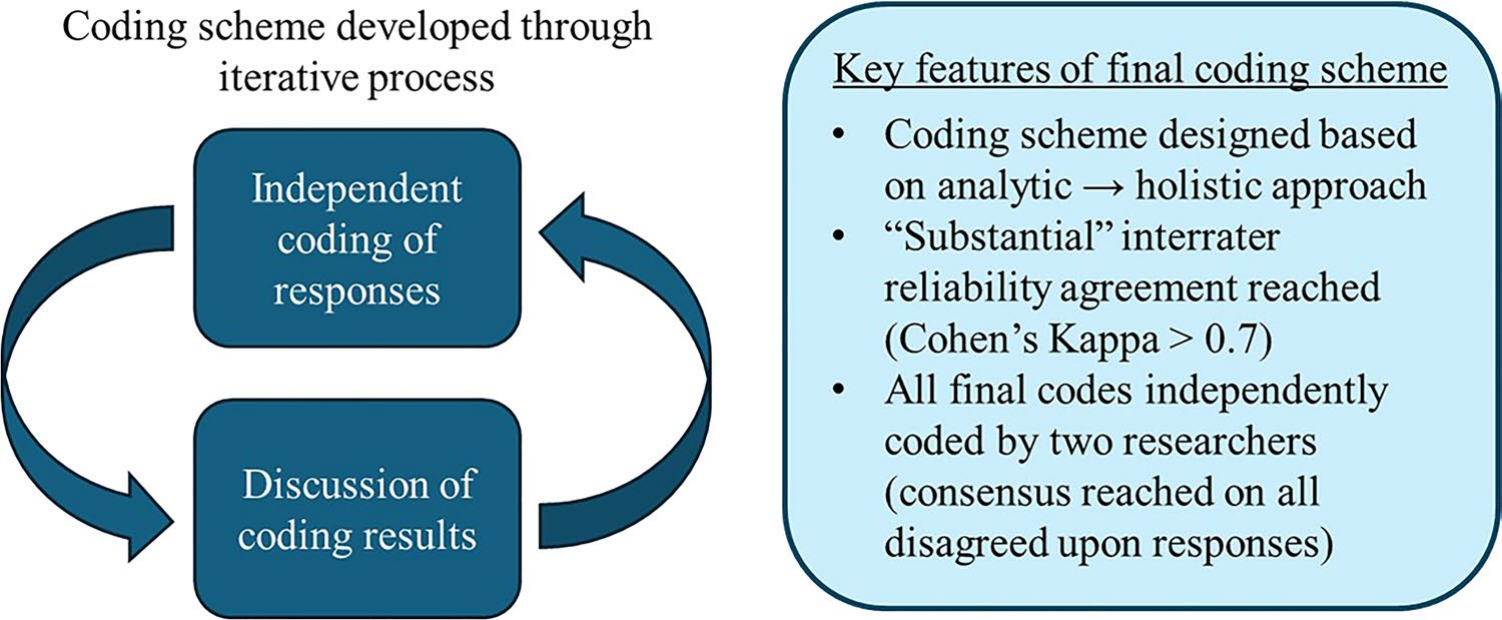
Visual summary of the process by which we developed our coding scheme and key features of our final coding scheme

To create the coding scheme, we conducted several rounds of independent analysis of the responses, each followed by a group discussion. Those discussions allowed us to identify what parts of the coding scheme worked well so we could make refinements as necessary. This was key to ensuring that we all agreed upon the definitions and characteristics of each code. We discuss this process further in the following paragraphs and show a summary of the iterative process in Fig. 2.

First, we familiarized ourselves with the data. We read through the students’ responses (the data) and identified patterns in the responses that aligned with the components of CMR. We discussed what characteristics of their responses would correspond to an explanation that was mechanistic, non-mechanistic, or somewhere in between. We created a series of analytic (i.e., non-mutually exclusive) coding categories that could be individually applied to identify the presence of each of the necessary components of a causal mechanistic explanation. Based on the presence or absence of the ideas captured by each of these analytic coding categories, we could assign an overall holistic code characterizing the CMR used. Others have found this “analytic to holistic” approach to coding scheme development valuable in analyzing complex concepts [75].

We identified four analytic coding categories: “Cause – Glucosuria,” “Mechanism – SGLT2 location,” “Mechanism – Decreased reabsorption,” and “Linking – Glucose needed for infection.” We define these coding categories and provide examples in the results (Table 1). We tested these analytic coding categories on another randomly selected set of 30 responses and found this new approach much more manageable. At this point, we were satisfied with our coding scheme and proceeded to attempt to establish inter-rater reliability (IRR). In the IRR process, two of us (RB and NB) independently coded the responses using the analytic coding categories. We then characterized the agreement between the coders by calculating Cohen’s kappa values for each analytic coding category. Cohen’s kappa is an agreement measure that considers the possibility of agreement occurring by chance [76, 77]. We pre-determined that reaching an initial agreement level above 0.7 between the two coders, corresponding to a “substantial” agreement, would be necessary to establish IRR [76]. In the first round of IRR, RB and NB independently coded a random selection of 39 responses. We increased the responses analyzed at this stage (from *N* = 30 to *N* = 39) to capture a wider range of student responses during the IRR process.

**Table 1:**
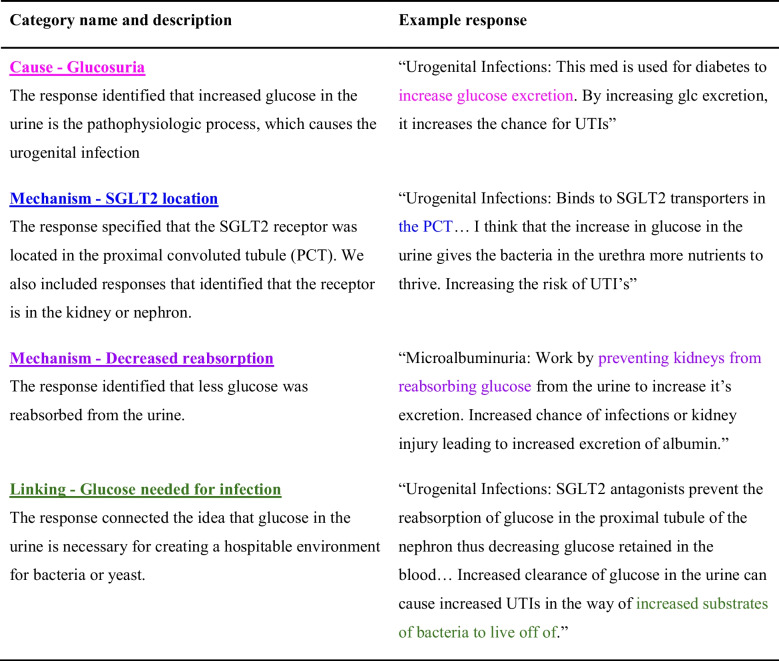
Descriptions and examples of the four analytic coding categories corresponding to the individual components of a fully causal mechanistic account of how and why an SGLT2 inhibitor would result in urogenital infections

After the first round of IRR, our agreement was above the Cohen’s kappa threshold (0.7) on three of the analytic codes: “Cause—Glucosuria,” “Mechanism—SGLT2 location,” and “Linking—Glucose needed for infection.” However, Cohen’s kappa value for the “Mechanism—Decreased reabsorption” coding category had only a “moderate” level of agreement (Cohen’s kappa = 0.557) and was below the 0.7 threshold [76]. After examining the responses coded differently in this coding category, we revised the code definition to ensure consistency. To be coded as “present,” the response must have discussed decreased glucose reabsorption related to the urinary tract instead of glucose reabsorption in other systems (e.g., the intestine). Additionally, it was sufficient for the response to discuss the “urinary tract” without specifying the exact location within that system (i.e., saying “nephron” without specifying the exact location of “proximal convoluted tubule”). These simplifications improved practical coding logistics while maintaining our ability to distinguish between types of causal mechanistic engagement.

**Fig. 3:**
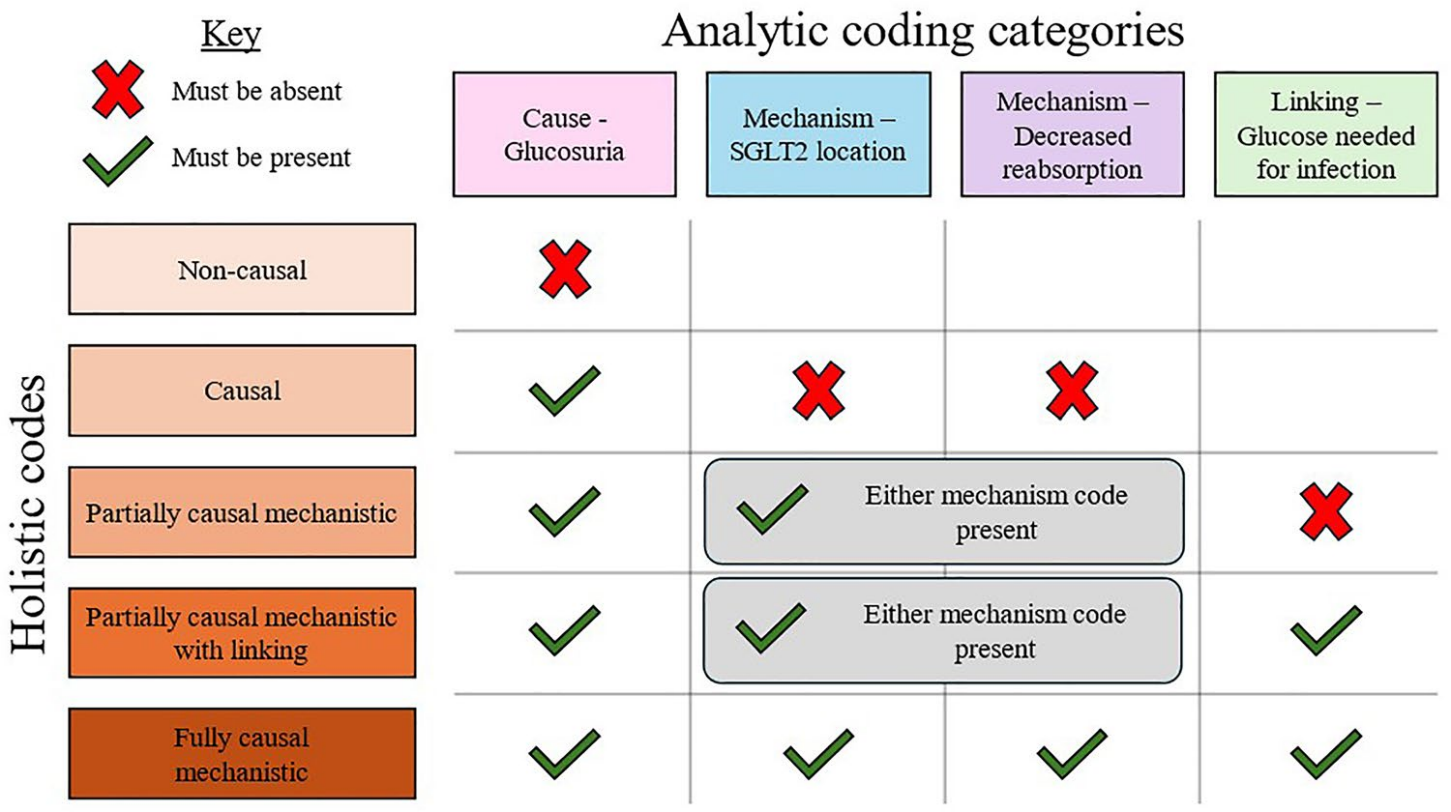
The combination of analytic codes (columns) that would produce each of the holistic codes (rows) characterizing the engagement in CMR. For the partially causal mechanistic and partially causal mechanistic with linking holistic codes, they must contain either “Mechanism-SGLT2 location” or the “Mechanism-decreased absorption” code

After making these changes, RB and NB independently coded all 88 responses. Cohen’s kappa for the initial agreement between the two coders on all the analytic coding categories ranged from 0.797 to 0.892, well above the 0.7 threshold. We resolved our disagreements and reached a final agreement upon a consensus code for any conflicting codes. We employed these final consensus codes to analyze the data and report the results of this manuscript. After coding the responses using the analytic coding scheme, we applied a series of logic statements to combine the different analytic coding categories into one of five holistic (i.e., mutually exclusive) codes, which characterized the overall degree to which the students engaged in CMR (Fig. 3).

### Data Analysis

To analyze the results, we conducted a series of Pearson’s *χ*^2^ tests of independence to assess the association between the different variables [78]. In some instances, the sample size was too small (more than 20% of cells had the expected counts less than 5) to perform the *χ*^2^ tests [79]. The *α* level was set at 0.05 for these statistical tests, but a Bonferroni-corrected *α* was used where appropriate [80]. For significant results, we calculated *φ* (or Cramer’s *V* where appropriate) to interpret the effect size of the significant result; values of 0.1, 0.3, and 0.5 correspond to small, medium, and large effect sizes, respectively [81]. We conducted all statistical tests using the statistical software SPSS version 28.0 (IBM SPSS Statistics for Windows, Version 28.0). Armonk, NY: IBM Corp—2021).

## Results

### Demographics

We invited 298 osteopathic medical students, the total number of enrolled students who had completed the standalone pharmacology course, to participate in the research. Of these, 203 (68%) initiated answering the questionnaires, with 88 (30%) students completing them and being included as participants. Of these, an almost equal number identified as men (50%) or women (49%), with 1% identifying as non-binary. Additionally, the bulk of the participants (83%) were under 30 years old.

### Students’ Experience with Pharmacology

All students surveyed were in their first or second year of medical school, with most participants in their second year (69%) of the program. All participants had completed their first-year pharmacology course at the time of the survey. There was no significant difference between the first and second years in selecting the correct multiple-choice answer (*χ*^2^ = 0.848, *p* = 0.357). At this university, students are ranked in quintiles. Students came from all five quintiles (9–19% each), though 23% did not know their quintile or preferred not to say. When asked about their prior pharmacology background, 33% declared relevant clinical experience, 33% had taken undergraduate pharmacology courses, and 22% reported no prior pharmacology experience.

### Resources/Strategies Medical Students Use to Study Pharmacology

We asked students to identify which resources/strategies they used to study pharmacology. This provided additional background information on the participants to give us a more complete picture of the ways in which they learned pharmacology. The top 5 most used resources/strategies were the coursepack (*N* = 87, 99%), Sketchy/Pictorize (*N* = 77, 88%), Anki/Quizlet/Flashcards (*N* = 71, 81%), watching other learning materials (*N* = 70, 80%), and First Aid (*N* = 61, 69%). A full breakdown of all the study resources/strategies used is included in Supplemental S1 ([71, 72, 82–84].

### Breakdown of the Potential SGLT2 Inhibitor’s Selected Adverse Effects

**Fig. 4:**
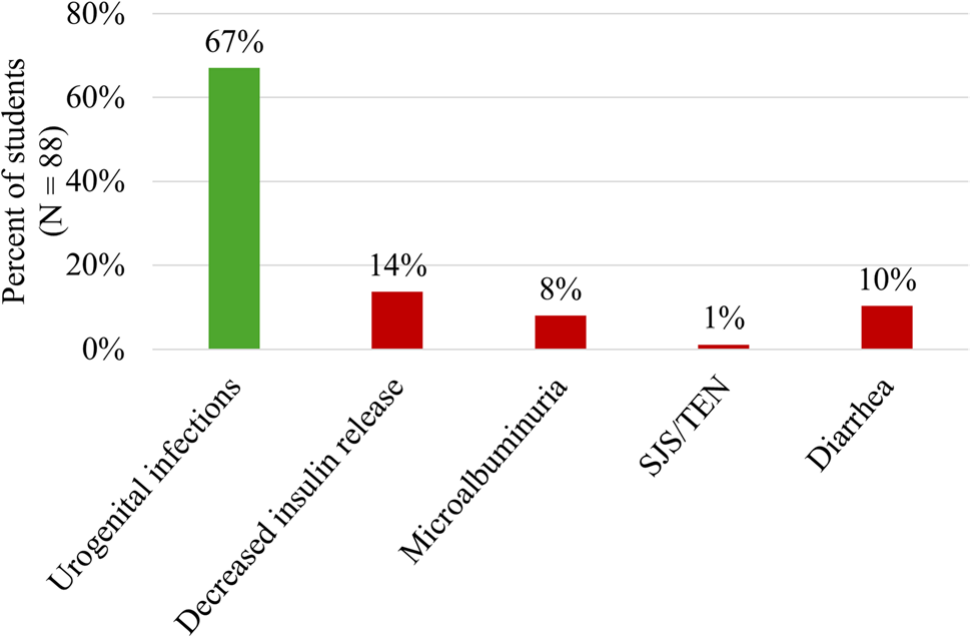
Breakdown of SGLT2 inhibitor selected adverse effects. Multiple-choice responses chosen by students (*N* = 88) for the adverse effect caused by an SGLT2 inhibitor. SJS/TEN, Stevens-Johnson syndrome/toxic epidermal necrolysis (TEN). MCQ: “The adverse effects of SGLT2 inhibitors involve which of the following? A. Urogenital infections; B. Decreased insulin release; C. Microalbuminuria; D. SJS/TEN; E. Diarrhea”

We conducted a one-sample chi-squared test to see if students selected any possible adverse effects more than others. We found that the majority of the students (67%) correctly identified that urogenital infections could be associated with SGLT2 inhibitors (Fig. 4), and this was the only response greater than 20% (i.e., the expected value if students selected all responses equally). The observed distribution of responses differed significantly from the expected distribution (*χ*^2^ = 125.409, *p*-value < 0.001).

### Emergent Patterns in Mechanistic Reasoning Used to Explain SGLT2 Potential Adverse Effects

**Fig. 5:**
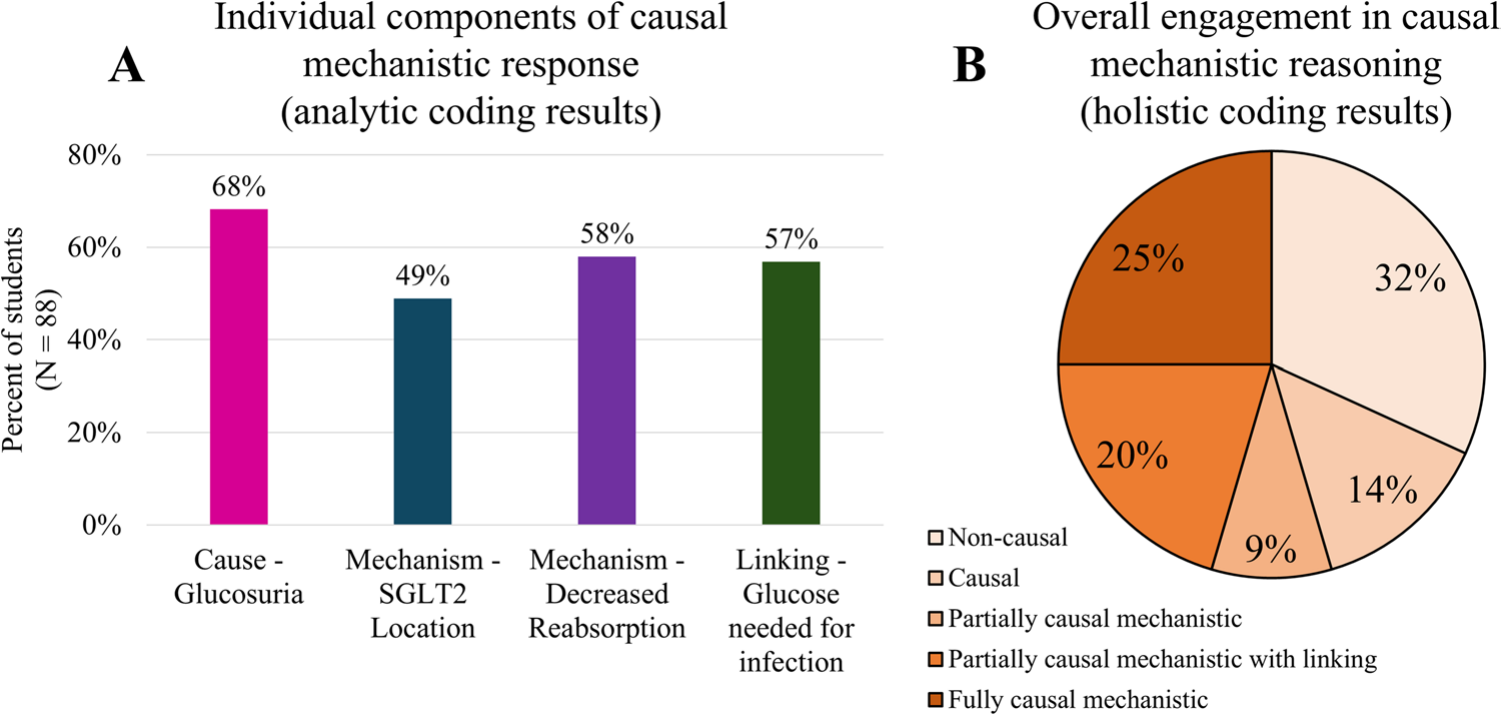
Components of causal mechanistic response. **a** Individual components of causal mechanistic response (analytic coding results): the percentage of students (*N* = 88) included each component of a causal mechanistic response according to our analytic coding scheme. **b** Overall engagement in causal mechanistic reasoning (holistic coding results). Based on the results from **a** and the logic statements described in Fig. 3, we determined the percentage of students (*N* = 88) engaged in different degrees of causal mechanistic reasoning

We analyzed the written responses and identified four analytic, non-mutually exclusive codes corresponding to different aspects of the causal mechanism by which SGLT2 inhibitors could cause a urogenital infection (Table 1). All four of the analytic codes were frequently identified in the students’ (49–68%) responses (Fig. 5a). The most common code was the “Cause—Glucosuria” category, in which students addressed that there would be increased glucose in the urine. However, when we translated the analytic codes to the holistic, mutually exclusive coding scheme (Fig. 3), we found that only a quarter of the students (25%) provided a fully causal mechanistic account of the phenomenon (Fig. 5b).

### Connection Between the Students’ Prediction of the Adverse Effect and Their Use of Mechanistic Reasoning

**Fig. 6:**
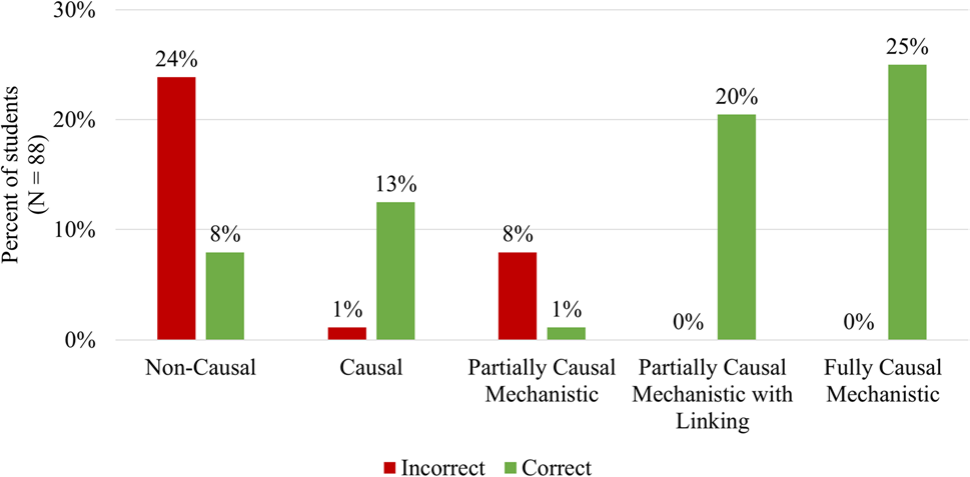
Engaging causal mechanistic reasoning. The percentage of students (*N* = 88) who engaged in each type of causal mechanistic reasoning (holistic coding scheme, Fig. 3) was separated by whether they got the multiple-choice question correct (green) or incorrect (red). Comparing those who correctly identified the adverse effect against those who did not, we found a significant difference in the distribution of the types of causal mechanistic reasoning used (*p*-value < 0.001, Cramer’s *V* = 0.799)

From the initial multiple-choice question, 67% of the students correctly identified that urogenital infections are the potential adverse effect caused by SGLT2 inhibitors (Fig. 6). To explore the relationship between engaging in causal mechanistic reasoning and correctly identifying the adverse effect, we used Pearson’s *χ*^2^ test of independence and found a statistically significant relationship of large effect size (*χ*^2^ = 56.129, *p*-value < 0.001, Cramer’s *V* = 0.799) (Fig. 6). Interestingly, of the 40 students who provided either a fully or partially causal mechanistic with linking response, all correctly identified the adverse effect (Fig. 6). As a post hoc analysis, we used a series of four *χ*^2^ tests (Bonferroni-corrected *α* = 0.0125) to explore which of the analytic codes were significantly associated with identifying urogenital infections as the adverse effect. We found there was a statistically significant positive association between correctly predicting the adverse effect and receiving the “Cause—Glucosuria” (*p* < 0.001, *φ* = 0.611) or “Linking—Glucose needed for infection” (*p*-value < 0.001, *φ* = 0.804) analytic codes. Based on the *φ* values, both associations had a large effect size. Neither the “Mechanism – SGLT2 Location” (*p* = 0.325) nor the “Mechanism – Decreased Reabsorption” (*p* = 0.197) codes were significantly associated with correctly identifying the adverse effect.

## Discussion

In this study, pre-clerkship medical students were prompted to identify and explain the occurrence of a potential adverse effect of an SGLT2 inhibitor. We hypothesized that the students who used CMR would present a more comprehensive understanding of how this drug affects the body and would, therefore, be more likely to predict the consequent adverse effect correctly. We found that the majority of the students (67%, Fig. 4) correctly predicted the adverse drug effect in MCQ. We analyzed the students’ responses to develop the coding scheme, converging CMR and pharmacology to determine the components necessary to explain, via short answer questions, how and why an SGLT2 inhibitor would lead to urogenital tract infections (UTI).

Understanding how this class of drugs develops this particular adverse effect (UTI) is directly related to knowledge of foundational biomedical sciences, such as the physiologic target for the drug to establish its therapeutic mechanisms, microbiology, and biochemistry. The students’ explanation must have identified the cause (glucosuria), unpacked mechanistic entities (receptor location and decreased glucose reabsorption), and linked these factors back to the adverse effect (glucose needed for bacteria growth). The resulting coding scheme captured those four key elements, and combining these codes provided an overall profile characterizing the degree to which students used CMR, revealing that only 25% provided a fully causal mechanistic explanation (Fig. 5b); this is in stark contrast with the results of the multiple-choice question in which the majority (67%) correctly predicted the adverse drug effect. However, our most exciting finding is that we found a significant association of a large effect size between using CMR and correctly predicting the adverse effect. Notably, *all* students who provided a fully causal mechanistic response correctly identified urogenital infections as the possible adverse effect (Fig. 3). This aligns with the findings from other studies, which have found mechanistic reasoning as associated with correctly predicting scientific phenomena [60, 66]. This further highlights how mechanistic reasoning can be a useful tool when encountering phenomena that might be unfamiliar to the learner [61]. Additionally, we found that the causal and linking components were significantly associated with making correct predictions, while the mechanistic details (the SGLT2 inhibitor’s location and function) were not. This could suggest that not all components of a causal mechanistic account are equally important toward the goal of developing productive predictions. It could be that the connective pieces (such as cause and linking) are particularly important. This idea could have important implications for broader research on mechanistic reasoning and warrants further study.

Our study is the first pharmacology medical education study to measure student use of CMR. While previous research in science education has explored CMR [47, 49, 50], our study uniquely focuses on pharmacology medical education, filling a significant gap in the literature.

Our findings align and support the previous science education conclusions mentioned in the introduction [57, 60, 66, 67], suggesting that similar benefits can be expected in medical pharmacology education. Our findings suggest that similar pedagogical strategies, as seen in biology, chemistry, and physics education, could be effectively applied in pharmacology, enhancing students’ ability to understand and predict pharmacological concepts in real-world scenarios. This is important as understanding the causal mechanisms underlying drug actions is crucial for medical students as it directly impacts their ability to make informed decisions in clinical practice [85, 86].

While our work is situated in the context of pharmacology education, educators in other disciplines may find the lens of mechanistic reasoning to be useful in their work. To do so, we recommend that first individuals reflect on the major phenomena in their discipline. Then, to reflect on what the components of a mechanistic explanation of those phenomena. So, what are the relevant underlying entities. Next, what properties and behaviors of those entities must be unpacked. Finally, how do the properties and behaviors link the underlying entities to the overall phenomenon. The goal is to identify relevant parts of this system that need to be discussed for students to engage in mechanistic reasoning. If done appropriately, students should be able to 'run' the system, predicting how the overall phenomenon would change in response to changes in the underlying entities or how the underlying entities would need to be changed to produce a specific result. This represents one of the most powerful aspects of mechanistic reasoning: it provides tangible ways to reflect on making productive changes in the classroom. For example, once the mechanism has been unpacked, educators can use that information to design activities centered around the phenomenon’s underlying entities, their properties, and how those entities are linked to the phenomenon. For those interested in reflecting more on how they can use the lens of mechanistic reasoning to support their work, we recommend reading the rich and robust work in science education on mechanistic reasoning [47, 49, 50].

## Limitations and Future Directions

The small sample size (*N* = 88) and low response rate (30%) limit the generalizability of our findings. However, the study was designed as an exploratory effort to examine the potential of CMR as a lens through which to understand students’ clinical thinking in pharmacology. Our study explored the use of CMR in only one pharmacological context (i.e., adverse drug effects). In addition, the assessment item used in this analysis was primarily a first-order recall question, which may limit the extent to which it captures deeper reasoning skills. While our findings indicate that students who employed full CMR answered the question correctly, the simplicity of the question may also explain why a majority of students (67%) responded correctly despite providing incomplete mechanistic explanations. Therefore, the high rate of correct answers may reflect students’ ability to recall key facts rather than using CMR per se. However, it is noteworthy that all students who demonstrated complete CMR in their responses answered the question correctly, supporting that CMR may enhance deeper understanding and accuracy in answering foundational clinical questions. Future research should incorporate higher-order, integrative assessment items, especially incorporating pharmacological and physiological knowledge, to more fully evaluate the added value of CMR in complex clinical reasoning.This includes studies exploring the applicability of our findings in other pharmacological contexts and with different groups of students, such as those in the clerkship.

Similarly, what students chose to include in their explanation was determined by the structure of this free response prompt (which was quite open-ended). Perhaps with additional scaffolding, more students will include more of the mechanism in their explanation. This is the tension of effective prompt design: designing the task in such a way that students understand the expectations for their answer (e.g., to include the mechanism in their response) without so much support that students can answer the question without thoughtful effort. For a more detailed discussion of the ways in which task design can elicit mechanistic explanations, please see Noyes et al. [87].

Although there is a theoretical foundation, there is no literature to provide such tools in the pharmacology context for practical learning education. Our current study offers exploratory insights into how students apply CMR when solving pharmacological problems without the explicit training from the course. Therefore, the results serve as a baseline of sorts—an exploratory study of the use of CMR in pharmacology to highlight the potential importance of this thinking strategy in this discipline. Future studies should investigate the impact of explicitly teaching CMR in pharmacology curricula.

While the present study focused on identifying spontaneous use of CMR in student responses, it remains unknown whether structured training in CMR would enhance students’ reasoning accuracy and depth, particularly when applied to higher-order, integrative clinical problems. Designing interventions that incorporate CMR frameworks, followed by assessments using complex pharmacological scenarios, would allow researchers to evaluate how well such instruction supports transfer of knowledge, improves clinical reasoning, and prepares students for board-style questions and real-world application. Given that nearly all the students (99%) used the coursepack associated with the curriculum to study pharmacology, this may be a productive place to incorporate principles of CMR to support student learning. The coursepack is a custom textbook designed by the faculty with content framed to follow the curriculum and the course learning objectives. Therefore, explicitly incorporating principles of CMR into the coursepack may not only provide a way for students to engage with mechanistic reasoning but faculty as well. This line of inquiry could inform the development of targeted pedagogical strategies to improve pharmacology education across health professions programs.

Recent advancements in educational technology have highlighted the potential of AI-driven tools to support and evaluate students’ feedback. According to Cooper and Klymkowsky (89), retrieval-augmented generation (RAG) AI systems leverage curated content to generate context-specific feedback, thereby reducing issues like hallucinations common in general AI models [88]. Based on our current findings, these AI systems may align with the goals of developing robust CMR skills in pharmacology education. These systems can provide real-time, individualized feedback to students, guiding them through complex reasoning tasks, allowing them to reflect on and refine their causal explanations. Learners can refine their understanding of pharmacological mechanisms. For instance, RAG-based chatbots can prompt students to elaborate on their reasoning, identify gaps in their explanations, and suggest areas for further study [88]. Instructors can also benefit from aggregated insights into common misconceptions and reasoning patterns within the class, allowing for targeted instructional interventions. Integrating AI tools into pharmacology education could enhance students’ ability to connect core concepts with clinical applications, fostering deeper learning and critical thinking skills. In another example, Ariely et al. (90) showed findings from a controlled experiment that demonstrated that a CM-based grading scheme can be used to identify meaningful patterns and inform the design of formative feedback that promotes students’ ability to construct explanations in biology [89]. Natural language processing and machine learning algorithms can evaluate student responses and provide feedback, enabling automated assessment and personalized learning in scientific writing for science education.

## Conclusion

Our results and strategic coding encourage other researchers and instructors to apply the framework of CMR to a broader range of core concepts both within medical pharmacology and in other closely related disciplines, as this foundational study provided promising results. Exploring student thought through the lens of CMR demonstrated a correlation with correctness and provided more insight into what resources students use in medical pharmacology. With complex phenomena, it can be challenging to identify which aspects of the system are the most important to the function of that system. Reflecting on the underlying entities most relevant to a pharmacological outcome, as well as the properties and behaviors of these entities that contribute to the observed effects, provides a structured framework for researchers. This approach can enhance understanding of how students engage in critical pharmacological reasoning and develop their learning processes. Ultimately, we encourage collaboration between educational researchers, pharmacology instructors, and curriculum developers to refine and expand upon our foundational findings.

## Supplementary Information

Below is the link to the electronic supplementary material.

Supplementary file1 (DOCX 171 KB)
