# Supplementary material for: Causal Mechanistic Reasoning as a Tool to Explore Medical Students’ Predictions of Pharmacology Phenomenon: Connecting Core Concepts with Clinical Applications
Source: Med Sci Educ. 2025 Jun 4;35(5):2329–42. doi: 10.1007/s40670-025-02432-6 (PMC12812137; doi:10.1007/s40670-025-02432-6)
Supplement: Supplementary file 1 — Supplementary file1 (DOCX 171 KB) [file 40670_2025_2432_MOESM1_ESM.docx]

**Supplemental Information**

**S1. Resources/strategies medical students use to study pharmacology**

We asked participants to indicate which resources/strategies they used to study pharmacology to gather a descriptive profile of the students in this study. These included several external resources, such as Sketchy [1], Anki [2], Pathoma [3], Pixorize [4], Boards & Beyond [5], Quizlet [6], and First Aid [7]. We clumped Anki and Quizlet together as they are both platforms for flashcards [2,6]. Sketchy and Pixorize were placed together as they are both platforms that use cartoon pictures to help with memorizing and learning content [1,4]. We also included one internal resource called OnTarget, an academic platform developed by our institutional specialist advisors and faculty in alignment with the American Osteopathic Association's (AOA) Commission on Osteopathic College Accreditation (COCA) requirements [8]. OnTarget offers resources to effectively and efficiently support pre-clerkship year students and maximize their learning experiences while navigating academic life. Figure S1 shows the full breakdown of these results.

**
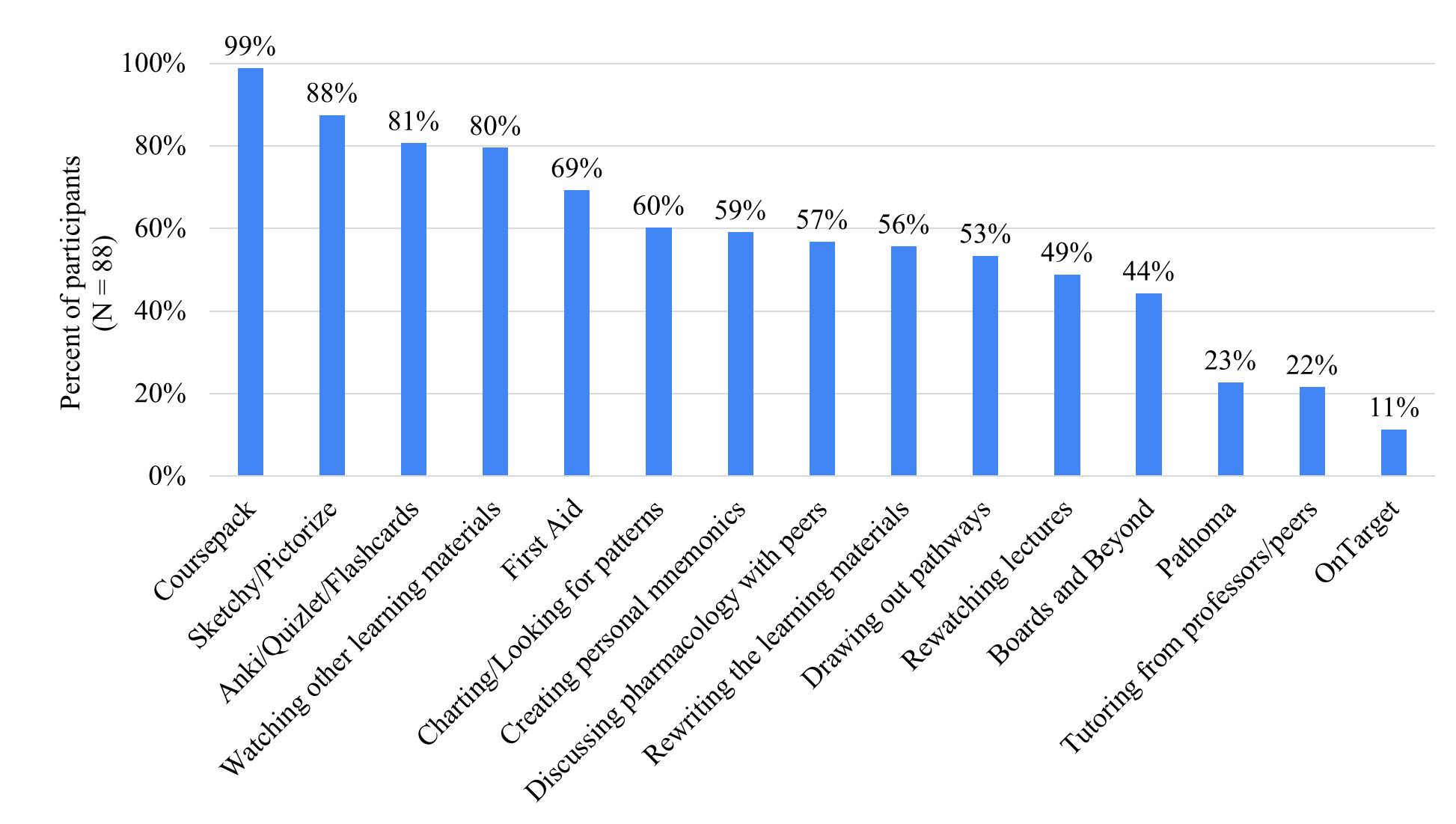
**

**Figure S1.** The resources/strategies students (N = 88) used to study pharmacology.

References:

1. Sketchy Medical [Internet]. Los Angeles, CA: Sketchy Group LLC; Available from: www.sketchy.com

2. Anki [Internet]. Available from: https://apps.ankiweb.net/

3. Sattar H. Fundamentals of Pathology [Internet]. 2022 Edition. Available from: pathoma.com

4. Pixorize [Internet]. New York, NY: Pixorize Inc.; Available from: www.pixorize.com

5. Boards&Beyond [Internet]. McGraw Hill; 2022. Available from: www.boardsbeyond.com

6. Quizlet [Internet]. San Francisco, CA: Quizlet Inc; Available from: www.quizlet.com

7. Sochat M, Le T, Bhushan V, Kaparaliotis P. First Aid for the USMLE Step 1. 31st ed. McGraw Hill; 2021.

8. Restini C, Silvestri S, Tobias C, Schwartz J, Armbruster A, Sadasivan M, et al. Impact of a Spiral Learning Strategy on the Integration of Pharmacology Content in a Pre‐clerkship Osteopathic Medical. The FASEB Journal. 2022;36:fasebj.2022.36.S1.R4286.
